# Social control drove ant evolution

**DOI:** 10.1073/pnas.2502664122

**Published:** 2025-03-24

**Authors:** Timothy A. Linksvayer

**Affiliations:** ^a^School of Life Sciences, Arizona State University, Tempe, AZ 85287

Variation across the ant tree of life reflects many independent evolutionary transformations from small and simple societies to huge and complex societies (1, 2). The most striking of these contain millions of workers and one or more reproductive queens, which collectively appear to act with unity of purpose (3). Caste-based reproductive division of labor also varies dramatically across ants, with some lineages having no morphological differences between reproductive and nonreproductive colony members, and other lineages having huge queens that are veritable egg-laying machines and miniature workers that are obligately sterile (4). In practice, this diversity across the ant tree of life provides tremendous opportunity to identify the causes and consequences of social group transformations that repeatedly occurred, which may help elucidate general mechanisms governing the elaboration of biological complexity. Recent studies using phylogenetic comparative approaches across ants are making exciting progress on these topics (1, 5–7). In PNAS, Matte and LeBoeuf (8) studied the evolutionary pathways to complex societies across ants, concluding that increasing social regulation of larval development promoted the elaboration of queen–worker dimorphism.

Matte and LeBoeuf (8) collected phenotypic data across the ant phylogeny for diet, larval morphology, and larval feeding behavior, together with the degree of queen–worker dimorphism and other measures of colony-level social complexity. They used a series of phylogenetic comparative methods to estimate the evolutionary correlation between these traits and then to determine which putatively causal models of trait evolution were best supported by the data across the ant phylogeny. Specifically, they first analyzed diet, larval feeding behavior, and larval morphology data from 99 species and showed that decreased larval neck length and loss of larval teeth were associated with less larval autonomy and increased dependence on adult nestmates. Next, Matte and LeBouef (8) used a larger dataset of 240 species with complete species-level data (as well as a dataset of 363 species, where genus averages were used in place of missing data) to study how diet and larval morphology were evolutionarily associated with queen–worker dimorphism, colony size, and worker polymorphism. Their best-supported evolutionary models suggest that transitions from semiautonomous active-feeding larvae to passive larvae reliant on portion-controlled food promoted the elaboration of queen–worker dimorphism and colony size. Matte and LeBouef (8) also identified shifts away from predatory diets as being a driver of larval trait evolution.

Researchers have hypothesized that the evolutionary elaboration of social complexity in social insects was driven by increases in colony size (2) or queen–worker dimorphism (4). Furthermore, the evolution of increasing queen–worker dimorphism is hypothesized to involve the elaboration of social regulatory mechanisms, including increasingly strict regulation of nutrition fed to individual larvae, involving the transfer of control of larval development and caste fate from individual larvae to their adult nestmates (9, 10). In PNAS, Matte and LeBoeuf’s results especially support this third hypothesis, suggesting that the evolutionary elaboration of queen–worker dimorphism, as well as colony size, required increasing social control of larval nutrition and development.

These empirical results are exciting because they identify specific larval and nurse traits shaping the nurse–larval interactions that regulate larval development and caste fate. In another new comparative study (6), LeBoeuf et al. study how trophallaxis, the social exchange of fluids among colony members, evolved across the ant tree of life. The origin of trophallaxis, which provides another mechanism of social interaction and social regulation of development, appears to also facilitate the elaboration of social complexity (6). Mechanistically, LeBoeuf et al. (11, 12) have also previously shown that fluids transferred among adult workers and between workers and larvae contain endogenously produced proteins, hydrocarbons, microRNAs, and juvenile hormone, a key developmental regulator (11, 12). Experimentally administering juvenile hormone to larvae affects the development time and size of the resulting adults (11, 13). Other researchers identified a previously undescribed social fluid produced during pupal molting, which contains nutrients, hormones, and neuroactive compounds, and is fed to larvae and increases larval growth and survival (14). These previously uncharacterized mechanisms of social control, together with additional mechanisms summarized by Matte and LeBoeuf (8), all provide nestmates with ways to influence and control the development and caste fate of brood. Variation in social regulation of larval development across ants that Matte and LeBoeuf (8) characterized is likely associated with variation in molecules transferred by adults to larvae. We can hypothesize that the transfer of control of development from larvae to their adult nestmates is associated with the elaboration of the queen- or worker-specific cocktail of gene products and other molecules administered to larvae (15, 16).

In PNAS, Matte and LeBoeuf (8) have carefully and creatively chosen traits to study that reflect the intensity of social regulation of larval nutrition and development. This nicely refocuses attention onto the types of fundamental social mechanisms that regulate development and the physiology of colony members and coordinate activity of colony members. Social insect research is usually at least superficially focused on understanding features that are directly or indirectly related to social insects’ unique social biology. However, social insect traits and genes are often implicitly or explicitly conceptualized in the absence of their social context (17). As a result, empirical approaches used to study these traits and genes often fail to consider social regulatory mechanisms. For example, caste dimorphism is often conceptualized simply as the plastic developmental response of larvae to variation in nutrition quantity and quality, ignoring the fact that variation in nutrition is often strictly regulated by adult nestmates, precisely as a mechanism that shunts larvae into alternate queen- or worker-developmental trajectories (9, 17). Similarly, nutrient-responsive molecular pathways that are found to be differentially expressed in queen- and worker-destined larvae are often considered to provide a complete explanation of the molecular mechanisms governing caste development, when in fact, gene products and other molecules produced and administered by nestmates feed into and regulate downstream developmental responses of larvae (11–13, 15, 16). A full mechanistic understanding of the regulation and evolution of social complexity associated with the transformation of small and simple to large and complex societies will require continued creative approaches to focus on traits and genes in the full context of social interactions.

In PNAS, Matte and LeBoeuf (8) studied the evolutionary pathways to complex societies across ants, concluding that increasing social regulation of larval development promoted the elaboration of queen–worker dimorphism.

Going forward, the comparative and mechanistic approaches discussed above will be further informed by forthcoming comparative genomic datasets containing hundreds of ant species. Comparative -omic studies will help to identify candidate genes and pathways that experienced shifts in regulation or protein sequence that are putatively associated with the evolution of social complexity and social regulatory mechanisms. Functional validation, especially focused on characterizing precisely how social regulatory mechanisms shape larval development, caste fate, and other aspects of colony function, will likely lead to especially exciting insight.

All comparative analyses, including those focused on trait evolution and comparative genomics, will greatly benefit from improved species-level ant phylogenies. While several previous studies of ant trait evolution were explicitly conducted at the genus-level with genus-level phylogenies across ants (e.g., ref. 1), Matte & LeBouef (8) and the other recent phylogenetic comparative studies described above (5, 6) used a set of “all-ant” phylogenies from 2018 that are also only based on phylogenetic information at the genus level (18). These phylogenies were created with a genus-level backbone tree based on DNA sequence from 673 species, where all remaining described ~14,000 ant species were then randomly grafted based on their genus name (18). As many traits vary strongly among closely related species, finer, ideally species-level resolution is important and will likely impact results. Relatively large species-level ant phylogenies do exist, inferred using DNA sequence data from over 1,700 species (7), and DNA sequence data are now available for many hundreds of additional species that can readily be added.

An additional powerful way forward that does not require all-ant species-level phylogenies is the use of species trees and trait data from carefully chosen model clades (19, 20). This approach is possible precisely because ants are so variable for many traits of interest, even within specific clades (e.g., a single genus or pair of sister genera). Careful and creative choice of study traits that can be readily quantified, together with focused mechanistic study of subsets of species can help to further elucidate how social regulatory mechanisms evolve.
